# Selective vulnerability of dopaminergic neurons in Parkinson’s disease connects *PRKN* and differential expression of *CHCHD2* and *GPNMB*

**DOI:** 10.1038/s41419-026-08926-4

**Published:** 2026-06-05

**Authors:** Franca Vulinovic, Arian Hach, Zied Landoulsi, Kerstin Tanzer, Daniel Alvarez-Fischer, Philip Seibler, Christine Klein, Patrick May, Aleksandar Rakovic

**Affiliations:** 1 Institute of Neurogenetics, University of Lübeck, Lübeck, Germany; 2 Luxembourg Centre for Systems Biomedicine, University of Luxembourg, Esch-sur-Alzette, Luxembourg; 3 Transversal Translational Medicine, Luxembourg Institute of Health, Strassen, Luxembourg

**Keywords:** Transcriptomics, Neurological disorders, Proteins

## Abstract

The mechanism(s) causing selective vulnerability of dopaminergic neurons in Parkinson’s disease (PD) remain largely elusive. To improve our understanding of mitochondrial involvement and related pathways suggested to play a role in this selective vulnerability, we used tyrosine hydroxylase (TH)-mCherry reporter-induced pluripotent stem cells generated by CRISPR/Cas9. We sorted neurons into pure TH-positive and TH-negative neurons upon differentiation into a dopaminergic neuron-containing cell culture. We characterized mitochondrial function in both dopaminergic and non-dopaminergic neurons from PD patients and controls and identified differentially expressed genes between patients and controls in both cell populations. Dopaminergic neurons had a lower mitochondrial membrane potential than non-dopaminergic neurons. Furthermore, ATP levels were lower in *PRKN* mutation carriers than controls, and mitochondrial mass was reduced in *PRKN* mutation carriers only in the TH-positive but not in TH-negative neurons. Importantly, in *PRKN* mutation carriers, we demonstrated elevated levels of dopamine, which can serve as a significant source of toxic, oxidized dopamine. Using unbiased RNA sequencing, we detected increased levels of *CHCHD2* and decreased expression of *GPNMB* in TH-positive neurons from Parkin mutation carriers compared to healthy controls. This suggests a possible interaction of these three PD genes in response to a dopaminergic neuron-specific increase in oxidative stress, which further leads to the selective vulnerability of dopaminergic neurons.

## Introduction

Parkinson’s disease (PD) is a progressive neurodegenerative disorder caused by the loss of dopaminergic neurons in the substantia nigra. Why dopaminergic neurons are preferentially affected remains a conundrum. The generation of induced pluripotent stem cells (iPSCs) from patient-derived blood cells or fibroblasts using cellular reprogramming techniques [1] has enabled the establishment of patient-specific in vitro disease models, including PD-relevant dopaminergic cultures, suitable for analyzing disease mechanisms at cellular and molecular levels [2]. However, the major challenge of studying PD in iPSC models is the lack of pure dopaminergic cultures. The existing differentiation protocols result in a mixed neuronal population with up to 20-30% dopaminergic neurons only [3, 4]. Thus, TH-reporter iPSC lines, which allow the sorting of TH-positive cells and the subsequent re-cultivation, may help to resolve this problem and better understand the selective vulnerability of dopaminergic neurons [5].

Approximately 15% of all PD patients have a genetic cause or significant genetic contribution [6]. Although less frequent than the more common idiopathic PD, well-characterized monogenic forms can serve as a valuable model for understanding the differential vulnerability of dopaminergic neurons. Mutations in PRKN cause the most common recessive form of PD (PARK-PRKN) [7]. We generated TH-mCherry reporter iPSC lines from two compound-heterozygous *PRKN* mutation carriers and a healthy control. During differentiation into dopaminergic neurons, the mCherry signal enabled the sorting of neurons into TH-positive and TH-negative populations using fluorescence-activated cell sorting (FACS), followed by re-culturing the sorted neurons for further analysis. Using this novel reporter model, we detected reduced mitochondrial protein levels exclusively in dopaminergic neurons of *PRKN* mutation carriers but not in TH-negative neurons. Finally, transcriptional analysis in pure dopaminergic versus non-dopaminergic neurons identified several differentially expressed genes and pathways between control and *PRKN* mutation carriers, revealing a novel link between *PRKN* and two other genes, i.e., *CHCHD2* [8]. and *GPNMB* [9]. implicated in PD pathogenesis.

## Materials and Methods

### Generation of mCherry reporter lines using CRISPR/Cas9

To generate the reporter lines, a CRISPR/Cas9 genome editing-based protocol was used to insert the coding sequence of mCherry or green fluorescent protein (GFP) directly downstream of the tyrosine hydroxylase (TH) gene, as previously described [5]. into the iPSCs from a healthy control and two PD patients carrying mutations in *PRKN* (Supplementary Table 1). Reprogramming of the parental iPSC lines was done by StemBANCC using Sendai virus. The parental iPSC lines from two *PRKN* mutation carriers (*PRKN* PD 1 (Del ex4 + c.823 C > T); *PRKN* PD 2 (c.823 C > T; c.1054 T > C)) and a healthy control (Control) were used to integrate the mCherry or GFP sequence into the TH locus, right before the stop codon.

In brief, iPSCs were electroporated using plasmids expressing both the Cas9 mRNA and the guide RNA (gRNA) targeting the nucleotide sequence of the TH gene immediately preceding the stop codon. In addition, cells were transfected with a “donor” plasmid carrying mCherry or GFP cDNA flanked by the sequences homologous to TH upstream and downstream of the TH’s stop codon. Upon transfections, iPSCs were plated on Matrigel-coated culture dishes and expanded in mTeSR1 (Stemcell Technologies) (in the first two days after transfection, supplemented with the ROCK inhibitor Y-27632 (10 µM)). The medium was changed daily until distinct colonies formed. Then, colonies were picked, and the correct integration of mCherry or GFP was analyzed by Sanger sequencing. To cultivate the correctly edited clones, cells were passaged every 5–7 days at a ratio of 1:4–6 by using 0.5 mM EDTA in PBS (Life Technologies). Cells were cultivated at 37°C and 5% CO2 in a humidified atmosphere.

### mRNA expression analyses using quantitative RT-PCR

According to the manufacturer’s instructions, total RNA from cell pellets was isolated using the RNeasy Mini Kit (Qiagen). 250 ng of RNA served as a template for reverse transcription (First Strand cDNA Synthesis Kit, Thermo Fisher Scientific). Quantitative real-time PCR (qRT-PCR) was performed on the Lightcycler 480 (Roche Diagnostics) using Maxima SYBR Green (Thermo Scientific). To confirm the pluripotency of iPSC reporter lines of patients and control, the mRNA expression of *GDF3* (F-primer/R-primer (5’-3’): AAATGTTTGTGTTGCGGTCA/ TCTGGCACAGGTGTCTTCAG), *NANOG* (F-primer/R-primer (5’-3’): TGAACCTCAGCTACAAACAG/TGGTGGTAGGAAGAG TAAAG), *OCT4* (F-primer/R-primer (5’-3’): CCTCACTTCACTGCACTGTA/CAGGTTTTCTT TCCCTAGCT) and *SOX2* (F-primer/R-primer (5’-3’): CCCAGCAGACTTCACATGT/CCTCCCAT TTCCCTCGTTTT) were analyzed. *TH* mRNA expression was also measured (F-primer/R-primer (5’-3’): CCCCCGACGCCACCACGCCAC/TGCAGCGGCCGCTGCTGCCAC. Beta-Actin (F-primer/R-primer (5’-3’): TGAAGTGTGACGTGGACATC/GGAGGAG CAATGATCTTGAT) was used as a housekeeping gene. Finally, mRNA expression of CHCHD2 and GPNMB was measured using the following primer pairs: F-Primer/R-Primer: GAAGTAATGCTGAGCCTGCG/TCATTAGGCCAATCCGTTTGC and F-Primer/R-Primer: TGCTGACTGTGAGACGAACC/TGCCATCCTTAAAGGCGAGG, respectively. Quantification was performed using the deltadelta-C_p_ (ΔΔC_p_) method.

### Evaluating colocalization of mCherry-positive and TH-positive neurons

The colocalization between mCherry-positive and TH-positive cells was examined by signal correlation. For this, 35-day-old neuronal cultures were co-immunostained using an antibody against mCherry (red) and TH (green). The colocalization of both wavelengths results in a yellow appearance of the neurons. The line scans were placed randomly over confocal images of neuronal cultures, and the intensities were measured via the “plot profile” option in Fiji for each channel. For each, Control TH-mCherry, *PRKN* PD1 TH-mCherry, and *PRKN* PD2 TH-mCherry, five randomly chosen line scans per confocal image were selected, and the Pearson coefficient r was determined. Statistical significance was determined via one-way ANOVA and corrected for multiple testing (Dunnett).

### RNA sequencing

RNA was extracted from iPSC-derived dopaminergic neuronal cultures and sorted into TH-positive and TH-negative neurons using FACS. Among these samples, three were extracted from TH-positive cells and three from TH-negative cells (one control and two different *PRKN* homozygous mutation carriers for each group). Illumina NextSeq2000 performed RNA sequencing. We utilized the Illumina DRAGEN RNA pipeline to process the RNA-Seq data [10]. DRAGEN includes an RNA-seq aligner and RNA-specific analysis components for gene expression quantification, using the following parameters: DRAGEN automatically detects the correct type of RNA-seq library, corrects for GC bias, and detects gene fusions. We used the gene annotation file hg38.ncbiRefSeq for quantification.

### Differential gene expression analysis

Raw count data for each cell type (TH-positive and TH-negative) were analyzed separately using the DESeq2 R package [11]. Genes with low expression (*n* < 10) were filtered out for each subset. Differential expression analysis was performed using a negative binomial generalized linear model with batch and disease status as covariates. Genes significantly differentially expressed (DEGs) were selected under the following parameters: |log2 Fold Change | ≥ 1, and FDR-adjusted *p*-value less than 0.05. We constructed a combined DESeq2 dataset that included TH-positive and TH-negative cells to assess the interaction effects between cell type and disease status. The design formula included batch, cell type, disease status, and their interaction ( ~ batch + cell_type + disease_status + cell_type:disease_status). The interaction term (cell_type:disease_status) was extracted to identify genes whose disease-related expression changes differed by cell type. Pathway enrichment analyses, including Gene Ontology (GO) [12]. and Kyoto Encyclopedia of Genes and Genomes (KEGG) [13]. Databases were conducted using the clusterProfiler package [14].

### Cell culture, differentiation, and cell sorting

Human iPSCs were cultivated in TesR1 medium (STEMCELL Technologies) on Matrigel (BD-Biosciences)-coated dishes. All iPSC reporter lines were fully characterized using immunocytochemistry and gene expression analyses.

iPSCs were differentiated into dopaminergic neurons using a published protocol [3]. To sort mCherry-positive and mCherry-negative neurons, cells were harvested at day 35 of differentiation and used for fluorescence-activated cell sorting (FACS) via FACS Aria III (BD Bioscience). Sorted mCherry-positive and -negative neurons were used for further analyses or were taken into culture for further maturation.

Human dermal fibroblasts were maintained in modified Dulbecco-Eagle medium (Thermo Fisher Scientific) supplemented with 10% fetal bovine serum (Thermo Fisher Scientific) and 1% penicillin/streptomycin (Thermo Fisher Scientific). Fibroblasts from four healthy controls without PRKN mutations and fibroblasts from four PD patients with *PRKN* mutations were used to examine GPNMB levels (P1 carries *PRKN*: [c.823 C > T]; [c.1054 T > C], P2 carries *PRKN*: [delEx1]; [c.924 C > T], P3 carries *PRKN*: P2 carries PRKN: [delEx4]; [c.924 C > T], and P4 carries PRKN: [c.1072Tdel]; [+delEx7]. The control and the patients are age- and sex-matched.

### Live cell imaging

TH-EGFP reporter iPSCs were differentiated into neuronal cultures on 25 mm round coverslips for all live-cell imaging experiments. Cells were transduced with a lentivirus expressing a Mito-DsRed fusion protein 48 h before imaging to study mitochondrial motility in iPSC-derived neurons [15]. To determine mitochondrial membrane potential, cells were treated with 100 nM TMRM (Thermo Fisher Scientific) for 30 min at 37 °C. To dissipate mitochondrial membrane potential, cells were treated with 10 µM Valinomycin. Before imaging, coverslips were mounted on an Attofluor Cell Chamber (Thermo Fisher Scientific). Mitochondria were imaged for three minutes at a frequency of 1 Hz as previously described [15]. All images were acquired using an LSM710 confocal laser microscope (Zeiss) with a Plan-Neofluar 40x/1.3 NA oil immersion objective (Zeiss).

### Immunocytochemistry and western blotting

iPSC and iPSC-derived neurons were fixed in 4% paraformaldehyde. Pluripotency of the iPSCs was confirmed using antibodies directed against OCT4 (1:500; STEMGENT), NANOG (1:200; STEMGENT), TRA-1–60 (1:200; STEMGENT), and SSEA4 (1:200; STEMGENT). iPSC-derived neurons were stained against TuJ1 (1:1000; Covance), TH (1:500; Millipore), and mCherry (1:500; Abcam). As secondary antibodies, goat anti-rabbit, Alexa 488, and goat anti-mouse Alexa 595 (1:1000, respectively, Invitrogen) were used.

For Western Blotting, protein concentration was determined using the Dc Protein Assay (BioRad). For SDS PAGE NuPAGE, 4-12% Bis-Tris gels (Life Technologies) were used. After gel electrophoresis, proteins were transferred to nitrocellulose membrane (Protran) and probed with primary antibodies against TH (1:1000; Millipore), GAPDH (1:30000; Cell signaling), MFN2 (1:2000; Abcam), TOM20 (1:3000; Santa Cruz), dopamine decarboxylase DDC (1:1000; Cell signaling), CHCHD2 (1:1000; Proteintech), and GPNMB (1:1000; Santa Cruz).

### Assessment of mitochondrial parameters

Cellular ATP synthesis was measured using the ATPlite kit (Perkin Elmer) according to the manufacturer’s instructions. The reporter lines of patients and a control were sorted at day 35 of differentiation, and the respective pellets of the mCherry-positive and mCherry-negative neurons were used for the ensuing analyses. Protein concentration was measured using the Pierce BCA protein Assay kit (Thermo Fisher Scientific). Comparisons between patients and the control were performed using an unpaired t-test. Statistical analyses were performed for 3-4 independent differentiations using GraphPad Prism 8.

The mitochondrial membrane potential was analyzed using the JC-1 dye (Thermo Fisher Scientific) according to the manufacturer’s instructions. In brief, reporter lines were sorted into mCherry-positive and negative neurons at day 35 and were replated on poly-L-ornithine/laminin-coated coverslips. On days 46–60 of differentiation, mCherry-positive and negative neurons from patients and the control were incubated in 1 µg/ml JC-1 for 30 min in the dark at 37 °C and 5% CO2 in a humidified atmosphere. Then, live-cell imaging was performed using an LSM710 laser confocal microscope (Zeiss). Confocal images were analyzed with Fiji ImageJ. Comparisons between patient and control samples were performed for both mCherry-positive and mCherry-negative cell populations using one-way ANOVA with Dunnett’s multiple comparisons test. Statistical analyses were performed for 2–3 independent differentiations using GraphPad Prism 8.

To analyze the amount of Reactive Oxygen Species (ROS) in control and patient-derived mCherry-positive reporter lines, we used CellRox Green (Thermo Fisher Scientific) according to the manufacturer’s instructions. In brief, neurons at day 50-60 of differentiation were incubated in 5 µM CellRox Green for 30 min at 37 °C and 5% CO2 in a humidified atmosphere, then fixed in 4% paraformaldehyde and mounted with DAPI. Cells were analyzed using an LSM900 laser confocal microscope (Zeiss), and at least ten randomly selected coverslip sections from two differentiations were used for quantification using Fiji ImageJ. For the per-cell analysis, the cellpose Python package was used to segment individual cells, followed by manually removing any malformed regions of interest [16]. Differences between patients and the control were assessed using Kruskal-Wallis and post-hoc Dunn tests. Test choices were determined via Shapiro-Wilk and Levene’s tests. The statistical analyses were performed using GraphPad Prism 8 and R. Differences between the measurements were considered significant at *p*-values < 0.05.

## Results

### TH-mCherry reporter lines enable the complete separation of TH-positive from TH-negative iPSC-derived neurons

**Fig. 1:**
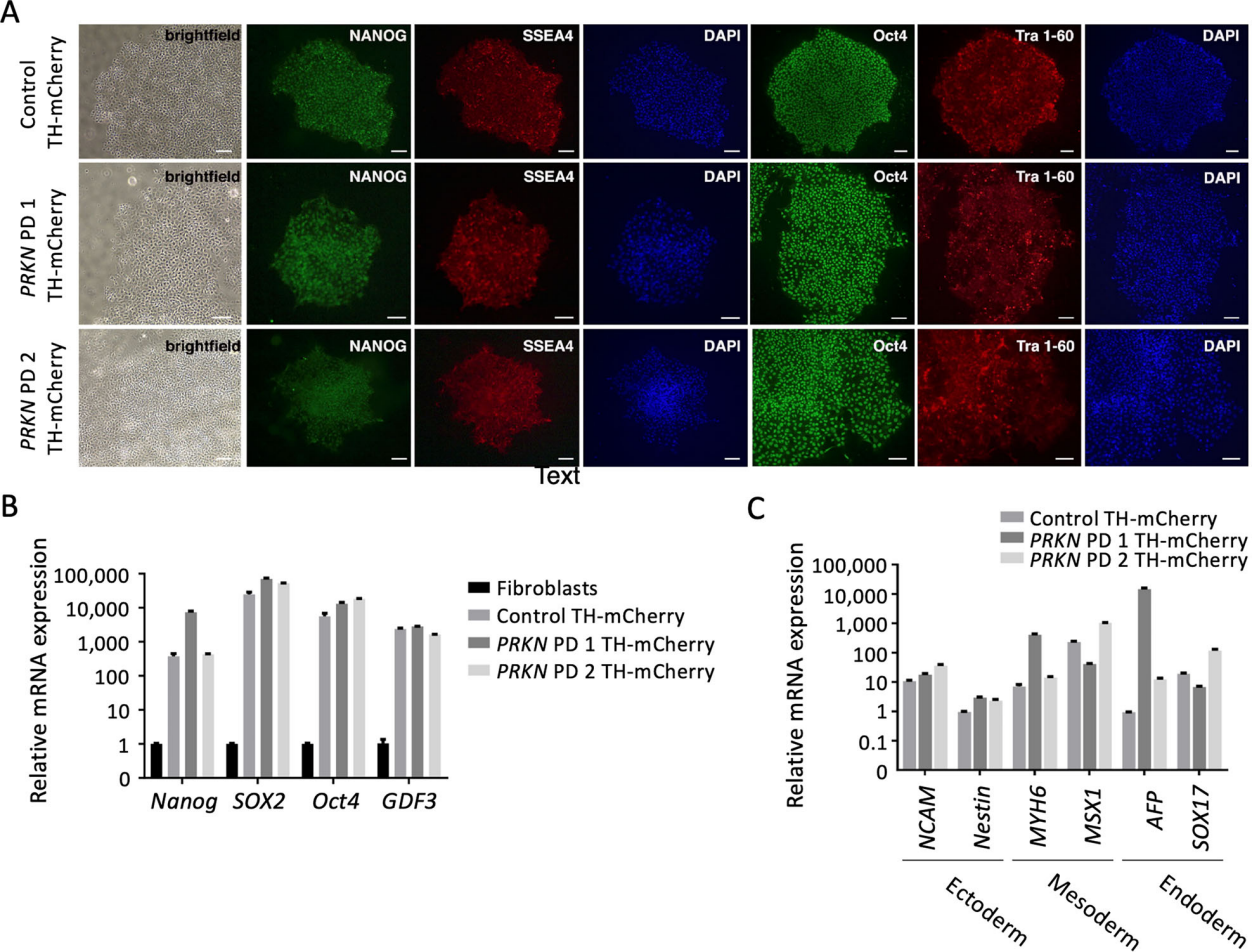
Pluripotency of TH-mCherry reporter iPSC lines. **A** TH-mCherry reporter lines of the healthy control (Control TH-mCherry) and two *PRKN* mutation carriers (*PRKN* PD1-THmCherry and *PRKN* PD2-THmCherry) express the pluripotency markers NANOG (green) and SSEA4 (red), as well as Oct4 (green) and Tra 1-60 (red). The nucleus was visualized using DAPI (blue). Scale bar: 100 µm. **B** Relative mRNA expression of the pluripotency markers *NANOG*, *SOX2*, *Oct4*, and *GDF3* is increased in TH-mCherry reporter lines compared to fibroblasts. *ACTB* was used as a reference gene. Data are expressed as mean ± SEM (*n* = 2). **C** Embryoid bodies of TH-mCherry reporter lines show mRNA expression of markers for the three germ layers (ectoderm: *NCAM*, *Nestin*; mesoderm: *MYH6*, *MSX1*; endoderm: *AFP*, *SOX17*). *ACTB* was used as a reference gene. Data are expressed relative to the respective iPSC line. Mean ± SEM (*n* = 2).

To generate TH-mCherry/GFP reporter lines, we used iPSC lines of two *PRKN* mutation carriers (*PRKN* PD 1 and *PRKN* PD 2) and a healthy control to integrate the mCherry or EGFP sequence within the *TH* locus [5]. Each line’s correctly edited iPSC clone was banked and used for in-depth characterization. All iPSCs displayed a stem cell-like colony-forming morphology of small cells with large nuclei (Fig. 1A). Immunofluorescence staining was performed to confirm the pluripotent state of the iPSCs, showing the expression of pluripotency markers *OCT4*, *NANOG*, *TRA-1–60*, and *SSEA4* in all cell lines (Fig. 1A). Accordingly, gene expression levels of *NANOG*, *OCT4*, *GDF3*, and *SOX2* were highly elevated in iPSCs compared to fibroblasts (Fig. 1B). Using embryoid body formation and subsequent gene expression analyses of markers of the ectoderm, endoderm, and mesoderm demonstrated the capacity of iPSCs to form cells of all three germ layers in vitro compared to iPSCs (Fig. 1C).

**Fig. 2:**
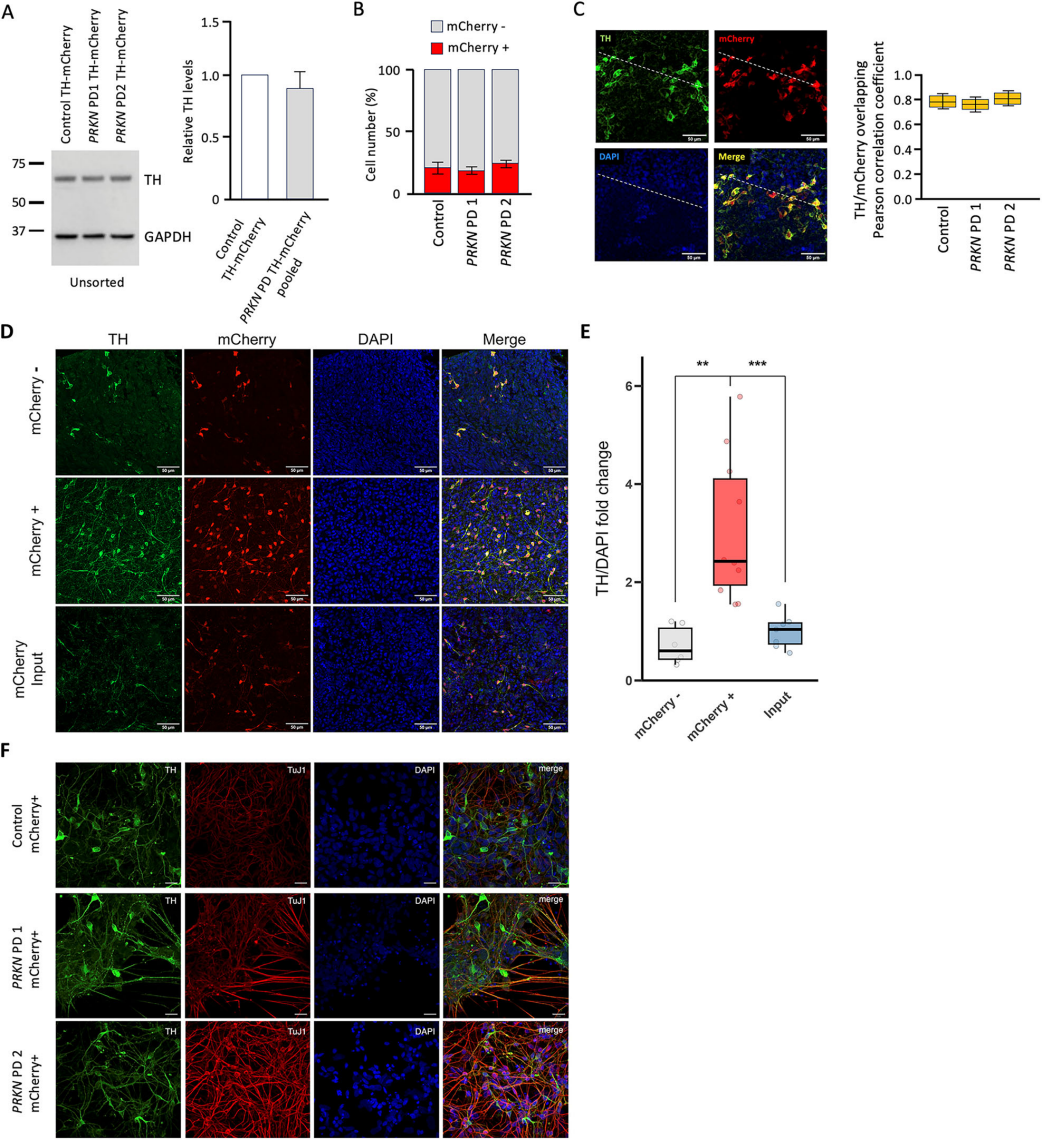
Sorting and recultivation of TH-mCherry reporter lines. **A** Western blot analysis of unsorted neuronal cultures at day 35 showed comparable TH protein levels between Control TH-mCherry, *PRKN* PD1 TH-mCherry, and *PRKN* PD2 TH-mCherry. **B** Sorting of TH-mCherry reporter line-derived neurons at day 35 of differentiation using FACS showed 21.6 (± 0.84)% of mCherry+ and 78.4 (± 0.84)% mCherry- cells. No difference in percentage of either mCherry+ or mCherry- neurons was detected between lines. **C** Immunostaining of non-sorted dopaminergic neuronal cultures showed complete overlapping between mCherry+ and TH+ neurons. The colocalization was quantified using Pearson’s correlation coefficient. Each line scan (white line) was placed randomly. The boxes show the quartiles of the dataset; the whiskers show the 1.5x of the interquartile range; the median values are indicated as horizontal lines inside the boxes; statistical analysis by one-way ANOVA and post-hoc Dunnett test. **D** Immunostaining of recultivated sorted mCherry- and mCherry+ neurons and non-sorted cultures (input) at days 45–50 of differentiation. **E** mCherry+ neurons were enriched compared to the input culture and mCherry- cells 3.06-fold and 4.25-fold, respectively. *n* = 6–10 randomly sampled coverslip sections of 1-2 differentiations. Scale bar: 50 µm. **F** Immunostaining of mCherry+ neurons at days 45–50 of differentiation. Neurons show expression of TH (green) and TuJ1 (red). The nucleus was visualized using DAPI (blue). Scale bar: 20 µm.

Next, the TH-mCherry and TH-EGFP reporter lines from the two *PRKN* mutation carriers and the healthy control were differentiated into dopaminergic neurons according to a previously published protocol [3]. On day 35 of differentiation, we analyzed TH levels in neuronal cultures. We found no difference in TH levels between Control and *PRKN* PD lines (Fig. 2A). Furthermore, the cells were sorted into mCherry-positive (mCherry + ) and mCherry-negative (mCherry-) neurons by FACS. As displayed in Fig. 2B, approximately 22% of the sorted cells were mCherry + . Importantly, we found no difference in the percentage of mCherry+ cells between *PRKN* mutation carriers and the control. In addition, we detected a complete overlap between mCherry+ and TH+ neurons in 50-day-old non-sorted neuronal cultures (Fig. 2C). Upon sorting, the neurons were successfully re-cultured and left to mature for an additional ten days before analysis. TH+ neurons were enriched 3.06-fold in mCherry+ sorted cells compared to non-sorted cultures and 4.25-fold compared to mCherry- sorted cells (Fig. 2D, E). Representative images of sorted and re-cultured mCherry+ neurons from a control and the two *PRKN* mutation carriers (*PRKN* PD 1 TH-mCherry and *PRKN* PD 2 TH-mCherry) are given in Fig. 2F. The sorted neurons at day 45-50 of differentiation exhibited distinct neuronal networks and expressed the dopaminergic neuronal markers TH (green) and TuJ1 (red).

**Fig. 3:**
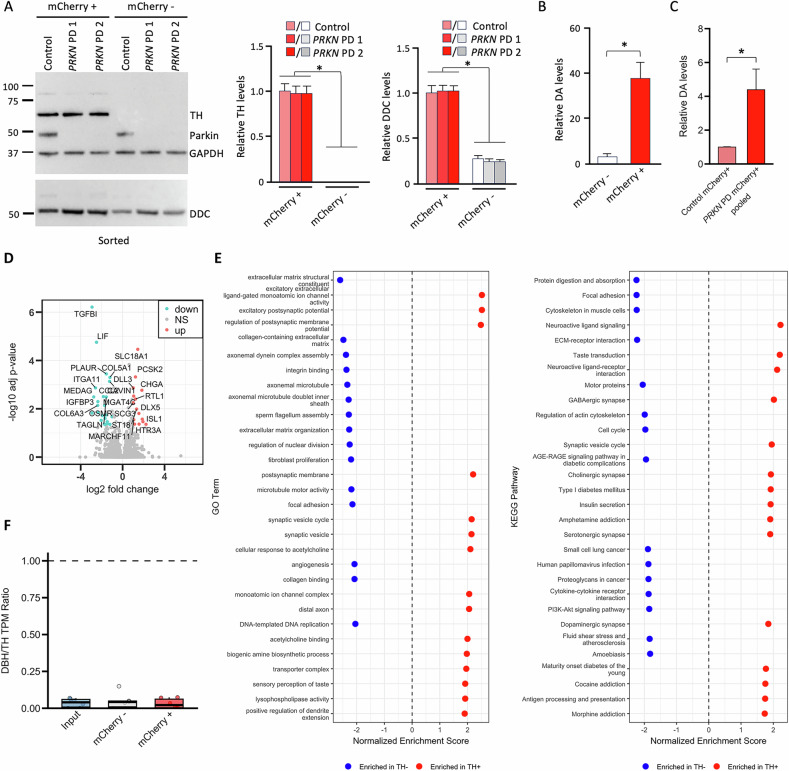
Dopamine synthesis in TH-mCherry reporter lines. **A** Western blot analysis of sorted mCherry+ and mCherry- neurons at day 50 of differentiation using antibodies against TH and DOPA decarboxylase (DDC). TH was only present in mCherry+ neurons. No difference in TH levels was detected between control TH-mCherry, *PRKN* PD1 TH-mCherry, and *PRKN* PD2 TH-mCherry. DDC was mainly present in the mCherry+ fraction, and no difference in DDC levels was detected between the lines. GAPDH protein levels were used for normalization. **B**, **C** Dopamine (DA) levels in sorted mCherry+ and mCherry- neurons were determined using HPLC. **B** Relative dopamine levels were higher in mCherry+ cells than in mCherry-negative cells, while **C** DA levels in mCherry+ neurons were significantly increased in *PRKN* PDs compared to control. **D** Volcano plot showing the differentially expressed genes in mCherry+ neurons compared to mCherry- cells. **E** Gene set enrichment analysis reveals dopaminergic neuron-specific pathway enrichment in mCherry+ neurons versus mCherry- cells. The left panel shows the most significantly enriched GO Biological Process terms. The right panel displays enriched KEGG pathways in mCherry+ neurons compared to mCherry- cells, determined by RNA sequencing analysis. **F** Dopamine β-hydroxylase (DBH) to TH transcript ratios expressed as transcripts per kilobase million (TPM) demonstrate differential noradrenergic marker expression across samples. Western blot experiments were repeated for three independent differentiations. RNA sequencing was performed for two independent differentiations, resulting in *n* = 6 samples per group. P values < 0.05 were considered significant.

To elucidate whether the differentiated and sorted mCherry+ neurons fulfill the characteristics of dopaminergic neurons by expressing the necessary proteins for dopamine synthesis, we checked the protein levels of TH and DOPA decarboxylase (DDC) using Western blotting (Fig. 3A). Upon sorting, TH was detected only in mCherry+ neurons, and no difference in TH levels was observed between control and *PRKN* mutation carriers (Fig. 3A). The expression of DDC was higher in mCherry+ than in mCherry- neurons, however, levels of DDC were comparable between control and *PRKN* mutation carriers (Fig. 3A).

Another key feature of dopaminergic neurons is their ability to synthesize dopamine. Therefore, we analyzed the dopamine content in sorted *PRKN* mutation carriers and control individuals using HPLC (Fig. 3B). Dopamine levels were higher in mCherry+ neurons than in mCherry- neurons (Fig. 3B). Comparison of mCherry+ neurons from *PRKN* mutation carriers with controls revealed significantly elevated dopamine levels in the *PRKN* mutation carriers (Fig. 3C), suggesting that increased dopamine levels may represent a significant source of toxic, oxidized dopamine. Differential expression and gene set enrichment analysis using RNA sequencing data from two independent differentiations of these cell lines demonstrate enrichment of neuron-specific GO terms and dopaminergic neuron-specific KEGG pathways in mCherry+ neurons compared to mCherry- cells, but no enrichment of noradrenergic neuron-specific pathways (Fig. 3D, E). Furthermore, transcripts per kilobase million (TPM) values were assessed to analyze potential noradrenergic contamination by calculating the dopamine β-hydroxylase to TH (DBH/TH) ratio per sample. DBH/TH ratios were consistently low ( ≤ 0.07) or zero for all mCherry+ sorted samples, further confirming the absence of noradrenergic neurons (Fig. 3F).

### Mitochondrial protein levels and mitochondrial function are altered in *PRKN* mutation carriers

**Fig. 4:**
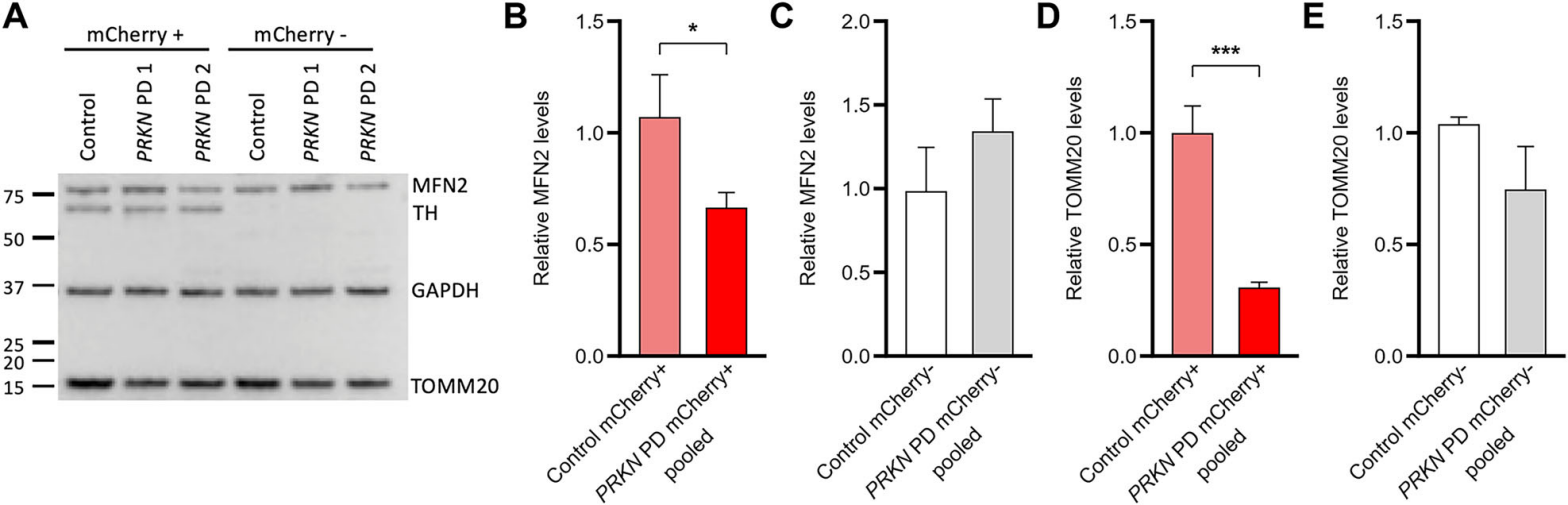
Mitochondrial protein levels are reduced in TH-positive *PRKN* mutants. **A** Western blot analysis of sorted mCherry+ and mCherry- neurons at day 50 of differentiation with antibodies against the mitochondrial proteins MFN2 and TOMM20. TH served as a marker for dopaminergic neurons, and GAPDH was used for normalization. **B** In TH-positive neurons, MFN2 protein levels were significantly reduced in *PRKN*-PD neurons compared to controls. **C** No differences in MFN2 protein levels were detected between lines in the TH-negative population. **D** TOMM20 protein levels were significantly reduced in TH-positive *PRKN* PDs compared to the TH-positive control. **E** No differences in TOMM20 protein levels between TH-negative control and *PRKN* PDs. All experiments were performed in three independent differentiations (*n* = 3-6 per group). Adjusted *p*-values < 0.05 were considered significant.

As PD is associated with disturbed mitochondrial function and Parkin is involved in mitochondrial quality control via mitophagy, we analyzed the levels of two mitochondrial proteins, i.e., Mitofusin 2 (MFN2) and Translocase of outer mitochondrial membrane 20 (TOMM20), using Western blotting, to gain insight into the mitochondrial mass and function in *PRKN* mutation carriers and control (Fig. 4A).

First, we checked the protein levels of MFN2, which is involved in mitochondrial fusion. In mCherry+ neurons, we detected significantly reduced protein levels of MFN2 in *PRKN* PD patients compared to the control (Fig. 4B). Moreover, there was no difference detectable between *PRKN* PD and control in mCherry- cells (Fig. 4C). Next, we analyzed levels of TOM20, another outer mitochondrial membrane protein, which were also significantly reduced in mCherry+ *PRKN* PD patients compared to the control (Fig. 4D) but not in mCherry- cells (Fig. 4E).

**Fig. 5:**
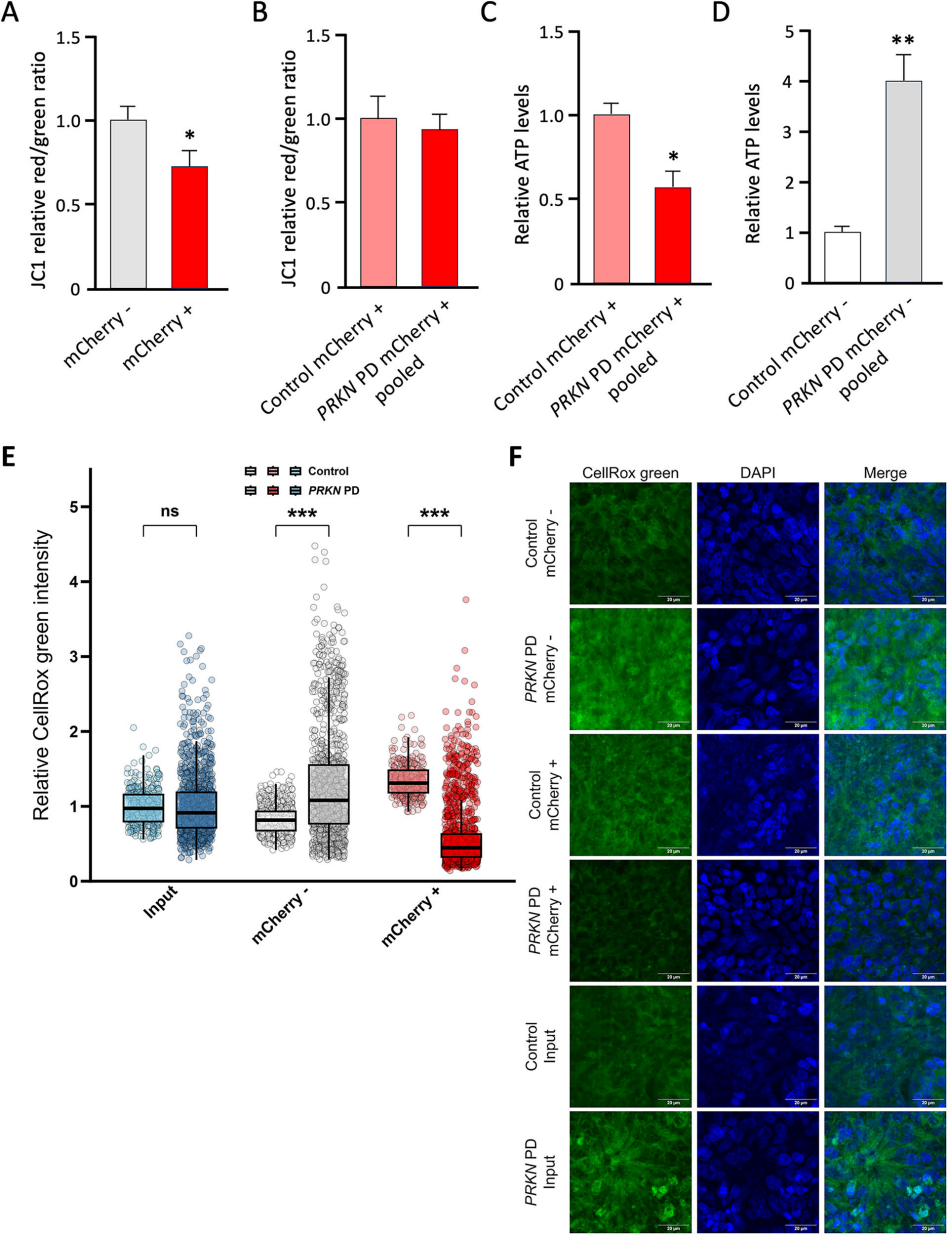
Mitochondrial function in TH-positive control and PRKN mutants. **A**, **B** Mitochondrial membrane potential (MMP) was measured in sorted and recultured mCherry+ and mCherry- neurons using the potentiometric dye JC1. **A** While mCherry+ neurons exhibit lower MMP than mCherry- neurons (**B**), no difference was observed between TH-positive control and *PRKN* PD neurons. **C** In TH-positive neurons, cellular ATP levels were significantly reduced in *PRKN* mutants compared to controls. **D** In contrast, in TH-negative neurons, cellular ATP synthesis was increased in *PRKN* mutants compared to controls. **E**,**F** Relative ROS levels highlight a disordinal interaction effect between sorting and disease status. Input cells showed no difference in ROS levels between *PRKN* mutants and controls. All experiments were performed in 2-3 independent differentiations (*n* = 2-6 per group). ROS levels were determined in *n* > 295 cells per group. Adjusted *p*-values < 0.05 were considered significant.

Having demonstrated that levels of specific mitochondrial proteins are altered in *PRKN* mutation carriers, we sought to elucidate whether neurons from *PRKN* mutation carriers also exhibit impaired mitochondrial function. One indicator of healthy mitochondria is their mitochondrial membrane potential (MMP). Therefore, we analyzed the MMP using JC-1 dye (Fig. 5A). Since all available potentiometric dyes used to assess MMP have their emission maximum at the same wavelength as mCherry, the cells were first sorted into mCherry+ and mCherry- neurons, then re-cultured, and left to mature for an additional 48 h before measuring MMP. The signal intensity of JC1 was significantly higher than the mCherry signal, allowing us to measure MMP in mCherry+ cells as well. In general, MMP was markedly lower in mCherry+ neurons compared to mCherry- neurons (Fig. 5A). However, in mCherry+ neurons, we found no difference between control and *PRKN* PD in terms of MMP levels (Fig. 5B). Although MMP was not impaired in *PRKN* mutation carriers, we still wanted to investigate whether mitochondrial function was impaired. Therefore, we quantified cellular ATP levels and found decreased ATP production in mCherry+ *PRKN* PD compared to control (Fig. 5C). To test whether this reduced ATP production is specific to Parkin deficiency and not dependent on cell type, we also examined mCherry- cells. Interestingly, we found increased ATP levels in *PRKN* PD patients compared to the control (Fig. 5D).

Dopaminergic neurons have a high energy demand. Due to their dopamine metabolism, which also generates reactive oxygen species (ROS), they may exhibit more significant oxidative stress levels than other cell types. To elucidate whether dopaminergic neurons carrying mutations in *PRKN* PD have altered levels of ROS, we analyzed a load of ROS using CellRox green in non-sorted, mCherry-, and mCherry+ neuronal cultures (Fig. 5E). In non-sorted neuronal cultures, ROS levels were comparable between *PRKN* mutation carriers and the control (Fig. 5F). In contrast, *PRKN* mutation carrier mCherry- cells exhibited a 1.56-fold increase in ROS levels when compared to the control. As with ATP levels, we observed opposing effects in mCherry+ cultures, where ROS levels were 2.23-fold lower in *PRKN* PD patient neurons compared to healthy control neurons. As ROS is a regular byproduct of mitochondrial respiration, reduced levels of ROS could be explained by impaired mitochondrial function in *PRKN* PD lines.

For proof of concept, in addition to the TH-mCherry reporter lines, we also generated TH-EGFP reporter lines using iPSCs from a healthy control. Since mCherry is significantly brighter than EGFP and allows reliable separation and microscopy of TH-positive and TH-negative neurons in this manuscript, we used only TH-mCherry reporter lines. However, TH-EGFP reporter lines have great potential for assays based on fluorescent dyes with a similar emission maximum to mCherry. Using live cell imaging of unsorted dopaminergic TH-EGFP neuron cultures, we were able to monitor mitochondrial transport and visualize MMP and in EGFP+ (dopaminergic) and EGFP- (non-dopaminergic) neurons (Supplementary Video S1 and S2).

### Differential gene expression analysis reveals *CHCHD2* and *GPNMB* as the most differentially expressed genes between control and *Parkin* mutants

**Fig. 6:**
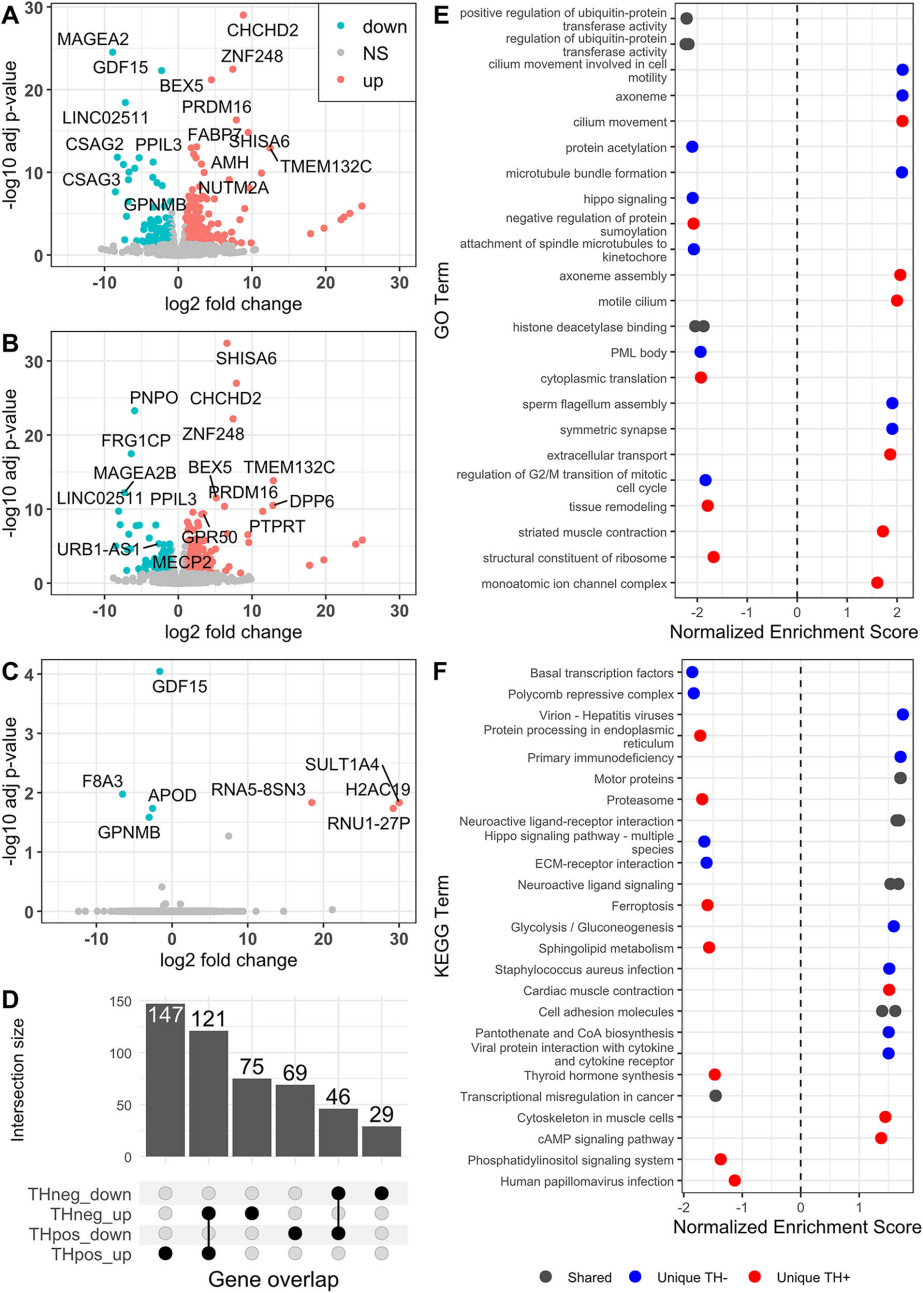
Differential gene expression analysis and GO term enrichment analysis of DEGs for *PRKN* mutation carriers vs. controls in TH-positive and TH-negative cells. **A–C** Volcano plot showing the relationship between the fold change and the associated p−value for each gene, respectively, in TH-positive (**A**), TH-negative cells (**B**), and the interaction between cell types and disease status (**C**). **D** Upset plot showing the intersection of upregulated and downregulated DEGs between TH-positive and TH-negative cells. **E** Most enriched gene sets among all significant genes (GO Biological Processes) and **F** KEGG pathways analysis in TH-positive, TH-negative, or shared between the two cell types. RNA sequencing was performed for two independent differentiations. Adjusted *p*-values < 0.05 were considered significant.

To identify candidate genes that are differentially expressed between control and *PRKN* mutation carriers in both TH-positive and TH-negative iPSC-derived neurons, we performed two independent, consecutive rounds of neuronal differentiations (further referred to as Differentiation 1 and Differentiation 2) followed by FACS into mCherry+ and mCherry- neurons. The samples from each differentiation round were sorted, and we proceeded with RNA sequencing. The RNA samples from mCherry+ and mCherry- neurons from *PRKN* PD1, *PRKN* PD2, and a healthy control were successfully sequenced and passed quality control. The principal component analysis (PCA) of Differentiation 1 revealed a clear separation between *PRKN* mutation carriers and a control (Supplementary Fig. 1A). We further compared the differential expression between control and *PRKN* PD patients in TH-positive and TH-negative cells. We identified 383 DEGs (adjusted *p* < 0.05 and absolute log2FC > 1) associated with PD in the TH-positive (Fig. 6A, D) and 271 DEGs in the TH-negative cells (Fig. 6B, D).

Of these, 191 DEGs were shared between the two cell types (Fig. 6D, Supplementary Data 1 for complete DEG list). Among the top ten most upregulated shared genes, only the *CHCHD2* gene was previously linked to PD. This gene serves as a mediator of oxidative phosphorylation, playing a crucial role in regulating electron flow within the mitochondrial electron transport chain [17]. Furthermore, we tested the interaction between cell types and disease status to determine whether the effect of PD differs between TH-positive and TH-negative cells (Fig. 6C). The results showed that eight genes were significantly upregulated or downregulated in PD, with more pronounced changes in TH-positive cells. Among these, *GPNMB* was among the most downregulated in TH-positive cells (Fig. 6A) but not in the TH-negative cells, and has been previously associated with PD. *GPNMB* variants are associated with PD risk [18]. Alterations in protein levels of *GPNMB* have been identified in PD and lysosomal storage disorders [9, 19]. The most significantly downregulated GO terms shared between TH-positive and TH-negative cells included ‘regulation of ubiquitin-protein transferase activity’ and ’positive regulation of ubiquitin-protein transferase activity’. In TH-positive cells (Fig. 6E), negatively enriched terms included ‘cytoplasmic translation’, ‘structural constituent of ribosomes’, and ‘negative regulation of protein sumoylation’. Upregulated terms included ‘cilium movement’, ‘axoneme assembly’, ‘motile cilium’, and ‘monoatomic ion channel complex’. TH-negative cells (Fig. 6E) exhibited enrichment in cell cycle control and developmental signaling pathways.

Shared KEGG pathways upregulated in PD patients included motor protein pathway and neuroactive ligand signaling (Fig. 6F). KEGG pathways exclusively downregulated in TH+ neurons included proteasome, protein processing pathways, ferroptosis, sphingolipid metabolism, and phosphatidylinositol signaling. Upregulated KEGG pathways in TH+ neurons included cardiac muscle contraction, the cytoskeleton in muscle cells, and the cAMP signaling pathway. TH- cells showed upregulation of several immune-related pathways and dysregulation of developmental control mechanisms. *PRKN* itself was not differentially expressed in *PRKN*-PD patients.

We performed real-time quantitative PCR with neurons generated during differentiation 2 to validate the RNA sequencing data. We confirmed the RNA sequencing data, showing increased levels of *CHCHD2* and decreased levels of *GPNMB* in *PRKN* mutation carriers compared to the control (Supplementary Fig. 1B). Aligning with the RNA sequencing data, we found no difference in *PRKN* mRNA expression between healthy controls and carriers of biallelic *PRKN* mutations. This may indicate a lack of nonsense-mediated decay of *PRKN* transcripts in certain mutation carriers (Supplementary Fig. 1C) as opposed to a complete lack of Parkin protein in the mutation carriers (Fig. 3A).

### *CHCHD2* protein was elevated explicitly in TH+ neurons from Parkin mutation carriers

A previous study demonstrated that GPNMB levels were increased in plasma samples from patients with idiopathic PD. Moreover, in cortical neurons derived from induced pluripotent stem cells, GPNMB was shown to regulate alpha-synuclein uptake [9].

**Fig. 7:**
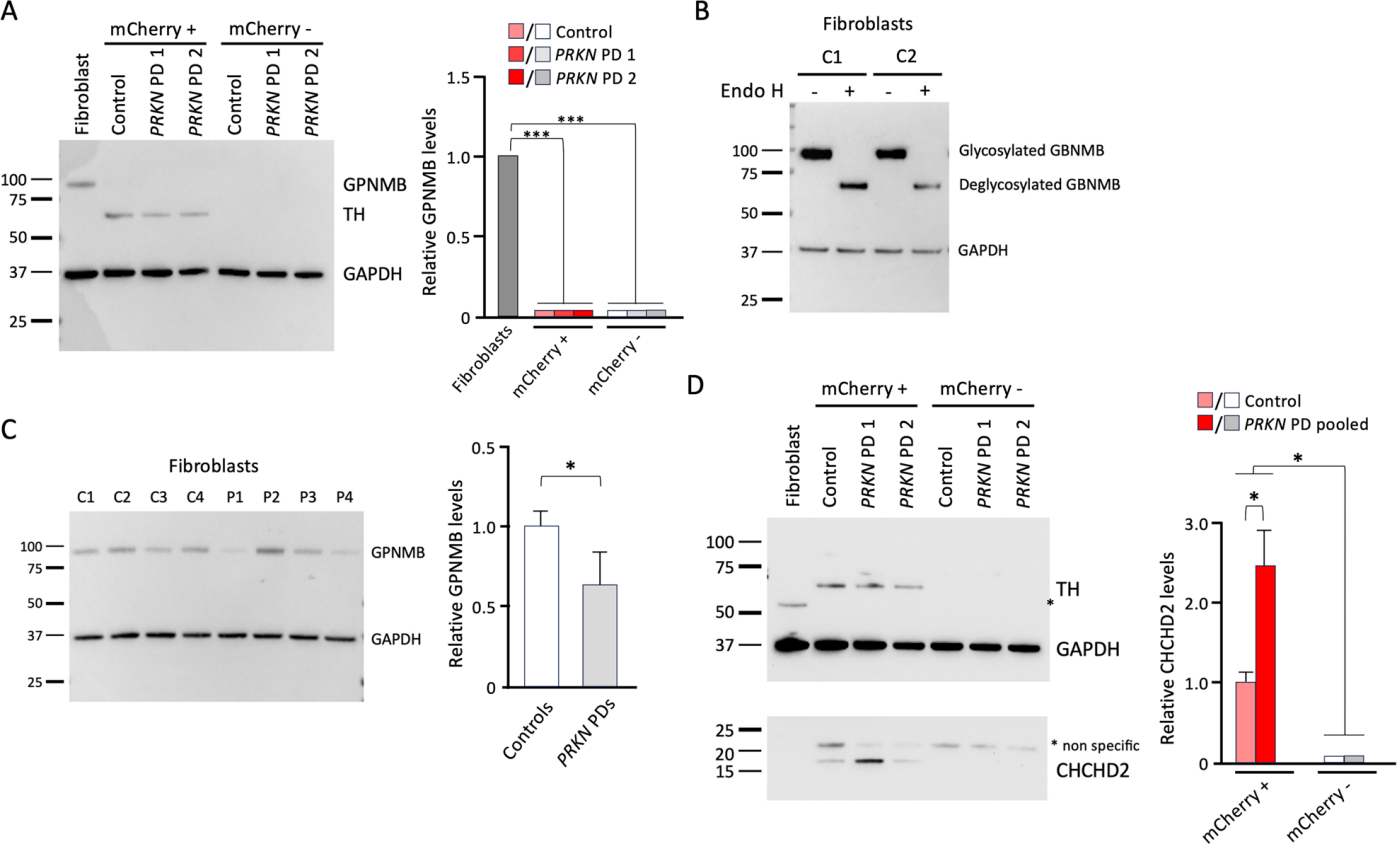
Protein analysis of GPNMB and CHCHD2 in iPSC-derived neurons and human dermal fibroblasts. **A** Western blot analysis of GPNMB in sorted mCherry+ and mCherry- neurons at day 50 of differentiation with an antibody against GPNMB. Additionally, a control fibroblast line was used. TH served as a marker for dopaminergic neurons and GAPDH as a loading control. **B** Western blot analysis of GPNMB in control fibroblasts (C1 and C2) treated with EndoH. GAPDH served as a loading control. **C** Western blot analysis of GPNMB in fibroblasts from four healthy controls (C1-C4) and PD patients with mutations in *PRKN* (P1-P4). **D** Western blot analysis of CHCHD2 in whole cell lysates using an antibody against CHCHD2. TH served as a marker for dopaminergic neurons, and GAPDH served as a loading control. *n* = 2-4. Adjusted *p*-values < 0.05 were considered significant.

Therefore, we examined the protein content of GPNMB in lysates from mCherry+ and mCherry- neurons by Western blotting. Additionally, protein lysate from primary human skin fibroblasts of a healthy control was used. Here, we detected GPNMB only in fibroblasts but not in either mCherry+ or mCherry- neurons (Fig. 7A). Given that GPNMB is a heavily glycosylated membrane protein, we validated the specificity of the previously employed anti-GPNMB antibody [9]. For this purpose, protein lysates from control fibroblasts were either untreated or treated with the deglycosylating enzyme Endo H and subsequently analyzed by Western blot. Treatment with Endo H resulted in a shift in the size of a GPNMB band to its predicted molecular weight, suggesting that the antibody is specific (Fig. 7B).

Because GPNMB is expressed in fibroblasts and is detectable by Western blotting, we compared GPNMB levels between four healthy control individuals and four patients carrying compound heterozygous *PRKN* mutations (Fig. 7C). Importantly, we detected lower GPNMB levels in *PRKN* mutation carriers compared to controls, confirming our RNA-Seq data.

A more recent study has shown that higher GPNMB levels were associated with a higher risk of PD [20]. However, mRNA expression and protein levels of GPNMB were enriched in oligodendrocyte progenitors rather than neuronal cells, which may explain the lack of detectable GPNMB protein in either mCherry+ or mCherry neurons in our study.

Finally, we measured the protein levels of CHCHD2 by Western blotting. We detected CHCHD2 only in mCherry+ neurons but not in mCherry- neurons or fibroblasts (Fig. 7D), which is consistent with previous publications showing that midbrain dopaminergic neurons have higher CHCHD2 levels compared with other types of neurons [21]. Importantly, CHCHD2 levels were higher in *PRKN* mutation carriers than controls, reflecting our RNA-Seq data.

## Discussion

Our cellular model represents a crucial improvement over existing dopaminergic neuron-containing iPSC-derived neuronal cultures, as it enables the separation of neurons of unknown identity from PD-relevant dopaminergic neurons [5, 22, 23]. Our previous work showed that homogeneous TH-positive neuron populations exhibit higher levels of electrical excitability [5]. a key physiological indicator of mature neurons, compared to TH-negative neurons [24]. Obtaining pure populations of TH-positive neurons is a fundamental step in understanding the selective vulnerability of dopaminergic neurons in PD. Indeed, using mCherry reporter lines, we demonstrated specific mitochondrial features of dopaminergic neurons, such as reduced mitochondrial membrane potential (MMP) compared to the currently used mixed neuronal cultures. Interestingly, we demonstrated differential expression of the PD-causative gene *CHCHD2* in pure vs. mixed dopaminergic neuronal cultures.

Differentiation of the TH-mCherry reporter iPSC lines into dopaminergic neurons, as described in a previously published protocol [3]. resulted in comparable numbers of mCherry-positive and mCherry-negative cells between control and mutants. As expected, the yield of mCherry-positive neurons was constantly at approximately 22%, underlining the importance of our cell model in generating pure cultures of dopaminergic neurons. Furthermore, as demonstrated in this study, we could re-culture the sorted cells at yields above 3-fold enrichment on average compared to non-sorted cells, allowing for further experiments with neurons forming distinct neuronal networks.

We confirmed typical protein expression for dopaminergic neurons, such as TH and DDC, which are both involved in dopamine synthesis and are equally present in *PRKN* mutation carriers and controls. RNA sequencing revealed a significant enrichment of dopaminergic neuron-specific pathways in TH+ neurons, ruling out noradrenergic contamination. In addition, significantly more dopamine was present in the dopaminergic neurons of *PRKN* mutants. It is tempting to speculate that increased dopamine levels can lead to its accumulation and oxidation into oxidized dopamine, leading to toxicity in dopaminergic neurons. Indeed, a previous study demonstrated that increasing dopamine levels in mice led to the accumulation of oxidized dopamine in the nigra of *DJ-1* KO mice, triggering the loss of dopaminergic neurons [25].

Several studies on inherited forms of PD caused by mutations in Parkin revealed a link between mitochondrial dysfunction and PD pathogenesis [26, 27]. We detected significantly decreased mitochondrial protein levels of both MFN2 and TOM20 in *Parkin* mutation carriers, exclusively in dopaminergic neurons, but not in the non-dopaminergic neurons. As previously described [28, 29]. Parkin is involved in the ubiquitination of MFN2 and TOM20 and their subsequent degradation. Thus, we expected increased levels of MFN2 and TOM20 due to impaired Parkin function. Nevertheless, a study using 6-hydroxydopamine (6-OHDA)-induced in vitro and in vivo PD models also showed decreased MFN2 levels [30]. suggesting that cells under dopamine-induced toxicity behave differently from non-dopaminergic cells with respect to Parkin-MFN2-TOMM20, which is consistent with our data. A similar trend was also observed in another study [31]. in which substantia nigra samples from idiopathic PD patients appeared to have reduced MFN2 expression. Additionally, studies on post-mortem substantia nigra samples from PD patients also revealed reduced TOM20 protein levels [32].

Our study additionally analyzed the mitochondrial membrane potential and mitochondrial function in dopaminergic neurons from *PRKN* mutation carriers compared to controls. Here, we demonstrated a lower MMP in dopaminergic neurons than in non-dopaminergic cells, which agrees with recently published studies [23, 24]. showing reduced MMP due to increased requirement for ATP and calcium to support the increase in electrophysiological activity over time. Nevertheless, their investigation, focusing on the effect of MMP treatment on dopaminergic neurons, could not detect any differences between control and *PRKN* mutation carriers, nor when comparing fibroblasts from *PRKN* mutation carriers and controls under basal conditions. However, in previous studies, treating cells with paraquat significantly reduced the mitochondrial membrane potential in mutants compared to healthy controls [33].

Referring to the mitochondrial function of synthesizing ATP, we found reduced ATP levels in dopaminergic neurons from *PRKN* mutation carriers compared to the control. These results confirm previous studies on iPSC-derived neurons and patient-derived fibroblasts [23, 33]. Interestingly, the non-dopaminergic neurons of *PRKN* mutation carriers showed increased ATP levels due to higher energy demands.

As previously reviewed [34]. oxidative stress is intensely involved in the dopaminergic neuronal loss in PD. Therefore, we analyzed ROS levels in our recultured dopaminergic neurons and non-dopaminergic cells under basal conditions. Surprisingly, we detected increased ROS levels only in non-dopaminergic cells from *PRKN* mutation carriers. In contrast, *PRKN* PD patient-derived dopaminergic neurons exhibited significantly reduced ROS levels compared to the healthy control. In this context, one crucial aspect to remember is that our data were obtained under basal conditions, and how mitochondrial parameters change under stress conditions remains elusive. Importantly, our results highlight the benefit of FACS-enrichment of patient-derived dopaminergic neurons in uncovering distinct cellular phenotypes otherwise masked by heterogeneous cell populations in non-sorted cultures. The higher basal energy demand and increased ROS levels observed specifically in non-dopaminergic cells of PRKN PD patients warrant further research to uncover the impact of other cell types on the ROS and energy demand-related vulnerability of dopaminergic neurons in these patients.

Our RNA sequencing analysis discovered increased levels of *CHCHD2* in *PRKN* mutation carriers compared to healthy controls. CHCHD2 is a mediator of oxidative phosphorylation, playing a key role in regulating electron flow in the mitochondrial electron transport chain [35]. It is tempting to speculate that *CHCHD2* levels were upregulated in Parkin mutant lines to buffer increased ROS production. Indeed, based on a previous study, *CHCHD2* is upregulated in response to mitochondrial stress [36].

In Drosophila, Chchd2 is required for neuronal integrity, and loss of pink1 or Parkin enhances Chchd28-8-associated neuronal defects [37]. suggesting that Parkin and CHCHD2 act synergistically to maintain the fitness of dopaminergic neurons. Based on our results, the deficit of functional Parkin in dopaminergic neurons is likely counteracted by the increase in *CHCHD2* expression.

The attenuation of ‘regulation of ubiquitin-protein transferase activity’ in both cell types suggests cell-type independent disruption of E3 ligase pathways relevant to *PRKN* function. However, TH+ neurons and TH- cells also showed markedly different responses to *PRKN* dysfunction. In TH-positive cells, the negative enrichment of ‘cytoplasmic translation’, ‘structural constituent of ribosomes’, and ‘negative regulation of protein sumoylation’ suggests selective protein synthesis dysfunction and altered post-translational regulation in addition to the shared disruption of E3 ligase pathways. The upregulation of terms like ‘cilium movement’, ‘axoneme assembly’, ‘motile cilium’, and ‘monoatomic ion channel complex’ in *PRKN*-PD TH+ neurons points to compensatory mechanisms to counter disrupted excitability and elevated mitochondrial stress [38].

TH-negative cells showed negative enrichment in cell cycle control and developmental signaling pathways, likely indicating reduced neurodevelopmental signaling. Notably, TH- cells showed upregulation of several pathways implicated in activated immune responses, suggesting that non-dopaminergic rather than dopaminergic cells drive pro-inflammatory responses that perpetuate selective vulnerability of dopaminergic neurons in PD.

Shared KEGG pathways upregulated in PD patients indicate systemic compensatory actions via the motor protein pathway and neuroactive ligand signaling. The disruption of proteasome, protein processing pathways, ferroptosis, sphingolipid metabolism, and phosphatidylinositol signaling exclusively in TH+ neurons highlights the pathways primarily involved in the selective vulnerability of these neurons to *PRKN* loss-of-function. Importantly, the disruption of proteostasis, ferroptosis, and sphingolipid metabolism has previously been linked to *PRKN*-PD or general PD pathophysiology [39–42]. Upregulated pathways in TH+ neurons indicate compensatory upregulation of pathways related to cytoskeletal integrity, calcium homeostasis, and neuroprotection via cAMP signaling. This suggests these neurons are mounting adaptive responses to maintain survival under pathological conditions.

Finally, we detected reduced *GPNMB* expression in dopaminergic neurons of *PRKN* mutation carriers compared to healthy controls. A previous study in inducible human iPSCs–derived cortical neurons showed that loss of GPNMB was accompanied by a marked reduction in a-Synuclein at the synapse [9]. While the authors used cortical neuronal cultures modified using CRISPR/Cas9, our study employed dopaminergic neuronal cultures from healthy control and PD patients carrying mutations in *PRKN*. The reduced levels of GPNMB may result from a compensatory mechanism or the existence of GPNMB-involving molecular pathways, serving as an alternative to GPNMB-induced a-Synuclein accumulation, which can lead to the selective vulnerability of dopaminergic neurons. A more recent study linked GPNMB to LRRK2 ^43^ suggesting that GPNMB is a valuable biomarker for PD.

This study has some limitations. First, our assessment of mitochondrial function lacks insight into oxygen consumption. Low total cell yields, plating requirements, and complex morphologies of the iPSC-derived neurons made their use in commonly available respiration assays challenging and did not yield reproducible results. Second, discrepancies between raw immunofluorescence-based TH/DAPI ratios and FACS measurements likely reflect methodological constraints of the applied immunohistochemistry protocol, such as antigen loss during permeabilization. Third, our findings are based on a limited number of cell lines, and validation in a larger number of edited hiPSC lines, including isogenic controls, would strengthen these findings.

In conclusion, we developed an iPSC-derived model of PD which i) allows studying pure dopaminergic neurons of *PRKN* mutation carriers; ii) is easily transferable to other PD-related genes; iii) has offered novel insights into the selective vulnerability of dopaminergic neurons through a link of mitochondrial dysfunction and elevated dopamine levels; iv) has identified a new and biologically plausible role of CHCHD2 and GPNMB in this process, and v) will likely be relevant not only to monogenic forms of PD but will have implications also for the common idiopathic PD, in the pathogenesis of which mitochondrial dysfunction is likewise known to play a significant role.

## Supplementary information

Supplemental video S1

Supplemental video S2

Reporting sumary

Supplementary videos

Supplementary Table 1

Supplementary data

Original blots

Supplemental information

## Data Availability

All other data generated or analyzed during this study are included in this published article and its supplementary information files. Requests for resources, raw data, or further information should be directed to and will be fulfilled by the corresponding author, Aleksandar Rakovic (aleksander.rakovic@uni-luebeck.de). This study did not generate new, unique reagents.
