# Supplementary material for: Selective vulnerability of dopaminergic neurons in Parkinson’s disease connects PRKN and differential expression of CHCHD2 and GPNMB
Source: Cell Death Dis. 2026 Jun 5;17(1):544. doi: 10.1038/s41419-026-08926-4 (PMC13241505; doi:10.1038/s41419-026-08926-4)
Supplement: Supplementary file 4 — Supplementary videos [file 41419_2026_8926_MOESM4_ESM.docx]

**Supplemental videos**

**Selective vulnerability of dopaminergic neurons in Parkinson’s disease connects *PRKN* and differential expression of *CHCHD2* and *GPNMB***

Franca Vulinovic, Zied Landoulsi, Arian Hach, Kerstin Tanzer, Daniel Alvarez-Fischer, Philip Seibler, Christine Klein, Patrick May, Aleksandar Rakovic*

**Video S1. Live-cell imaging of mitochondrial motility in dopaminergic and non-dopaminergic iPSC-derived neurons using TH-EGFP reporter lines.**

Time-lapse microscopy of unsorted TH-EGFP reporter iPSC-derived dopaminergic neuronal cultures transduced with lentiviral particles expressing Mito-Dsred.

**Video S2. Live-cell imaging of mitochondrial motility and mitochondrial membrane potential in dopaminergic and non-dopaminergic iPSC-derived neurons using TH-EGFP reporter lines.**

Time-lapse microscopy of valinomycin-induced mitochondrial membrane potential (MMP) degradation in an unsorted TH-EGFP reporter-IPSC-derived dopaminergic neuronal culture. Cells were treated with TMRM 30 minutes prior to imaging. Valinomycin was administered two to three seconds after the start of the video. The video is played at 15x speed. The loss of TMRM signal can be seen after 15 seconds of the video. Loss of mitochondrial membrane potential occurs first in TH-positive neurons, followed by loss of MMP in TH-negative cells. This is consistent with our data showing lower MMP in TH-positive neurons compared to TH-negative neurons.
