# Supplementary material for: Selective vulnerability of dopaminergic neurons in Parkinson’s disease connects PRKN and differential expression of CHCHD2 and GPNMB
Source: Cell Death Dis. 2026 Jun 5;17(1):544. doi: 10.1038/s41419-026-08926-4 (PMC13241505; doi:10.1038/s41419-026-08926-4)
Supplement: Supplementary file 5 — Supplementary Table 1 [file 41419_2026_8926_MOESM5_ESM.docx]

**Supplementary table 1 hiPSC lines.**

| **ID** | **AAO** | **AAE** | **Sex** | **Diagnosis** | **Gene** | **Variant** | **Zygosity** | **EbiSC ID** |
| --- | --- | --- | --- | --- | --- | --- | --- | --- |
| SFC818 | 15 | 57 | Male | Y | *PRKN* | delEx4 + c.924C>T | Compound het | NA |
| SFC821 | NA | 35 | Female | Y | *PRKN* | c.823C>T; c.1054T>C | Compound het | NA |
| SFC084 | NA | 63 | Female | N | Healthy control | NA | NA | STBCi033-A |

AAO = age at onset; AAE = age at examination; EBiSC = European Bank for induced pluripotent Stem Cells
