# Supplementary figures and images for: Selective vulnerability of dopaminergic neurons in Parkinson’s disease connects PRKN and differential expression of CHCHD2 and GPNMB
Source: Cell Death Dis. 2026 Jun 5;17(1):544. doi: 10.1038/s41419-026-08926-4 (PMC13241505; doi:10.1038/s41419-026-08926-4)

Figure 2A


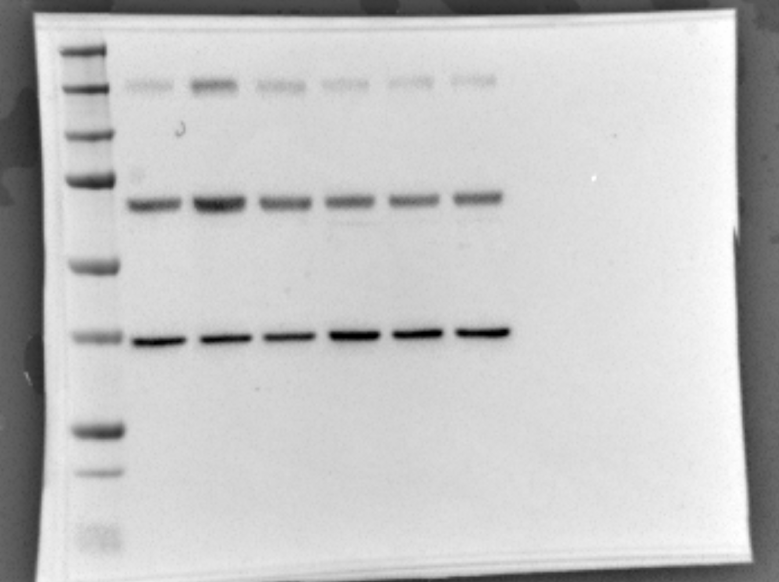


Figure 3A upper panel


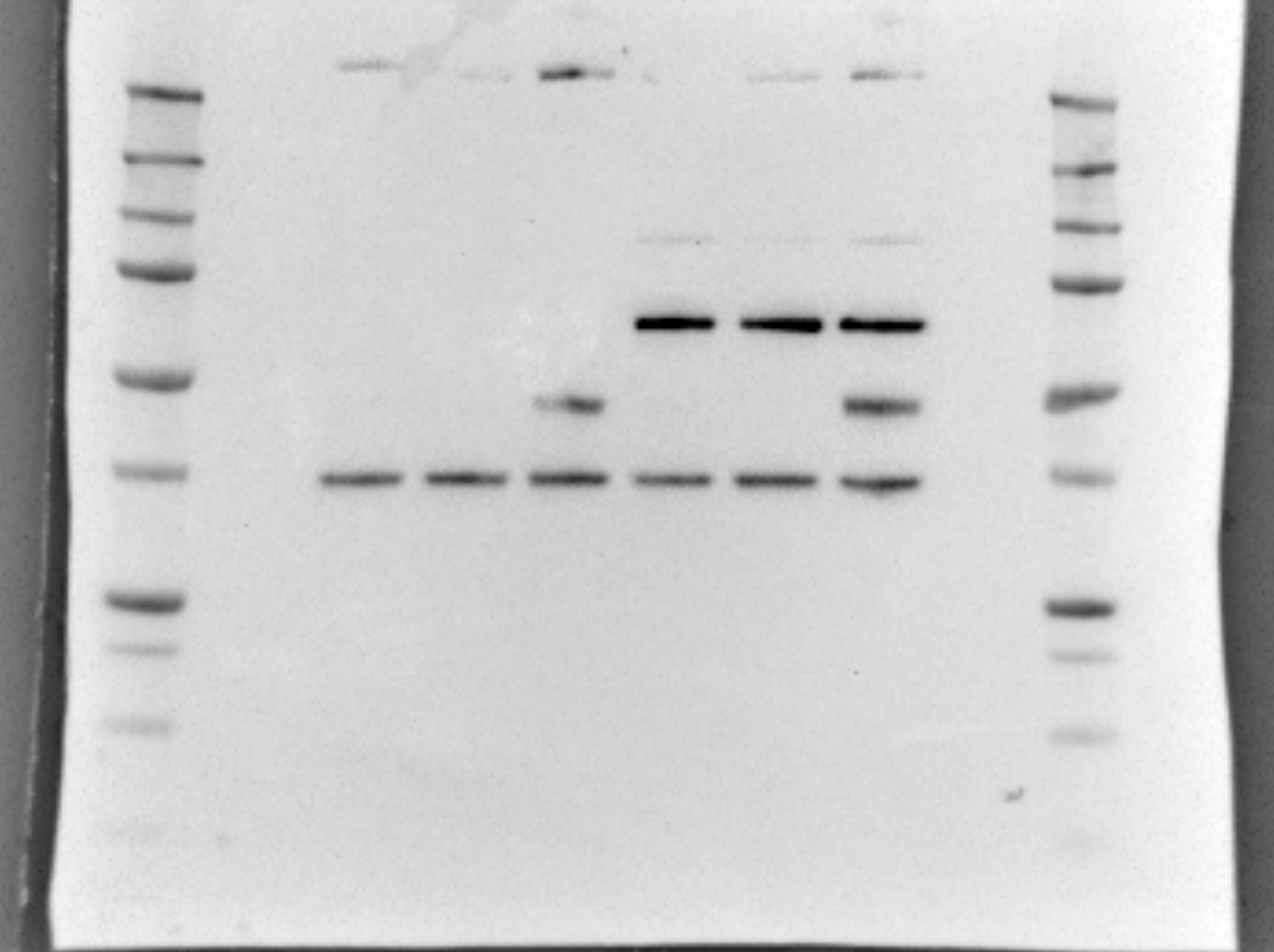


Figure 7A


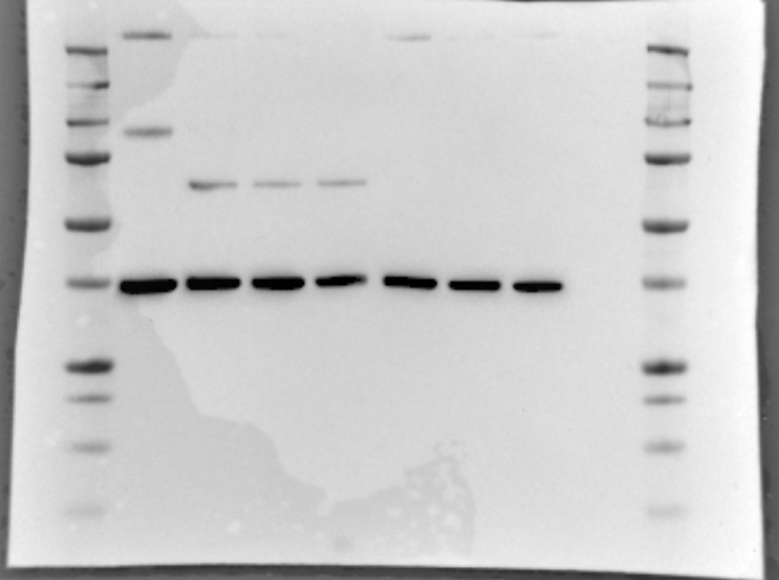


Figure 7B


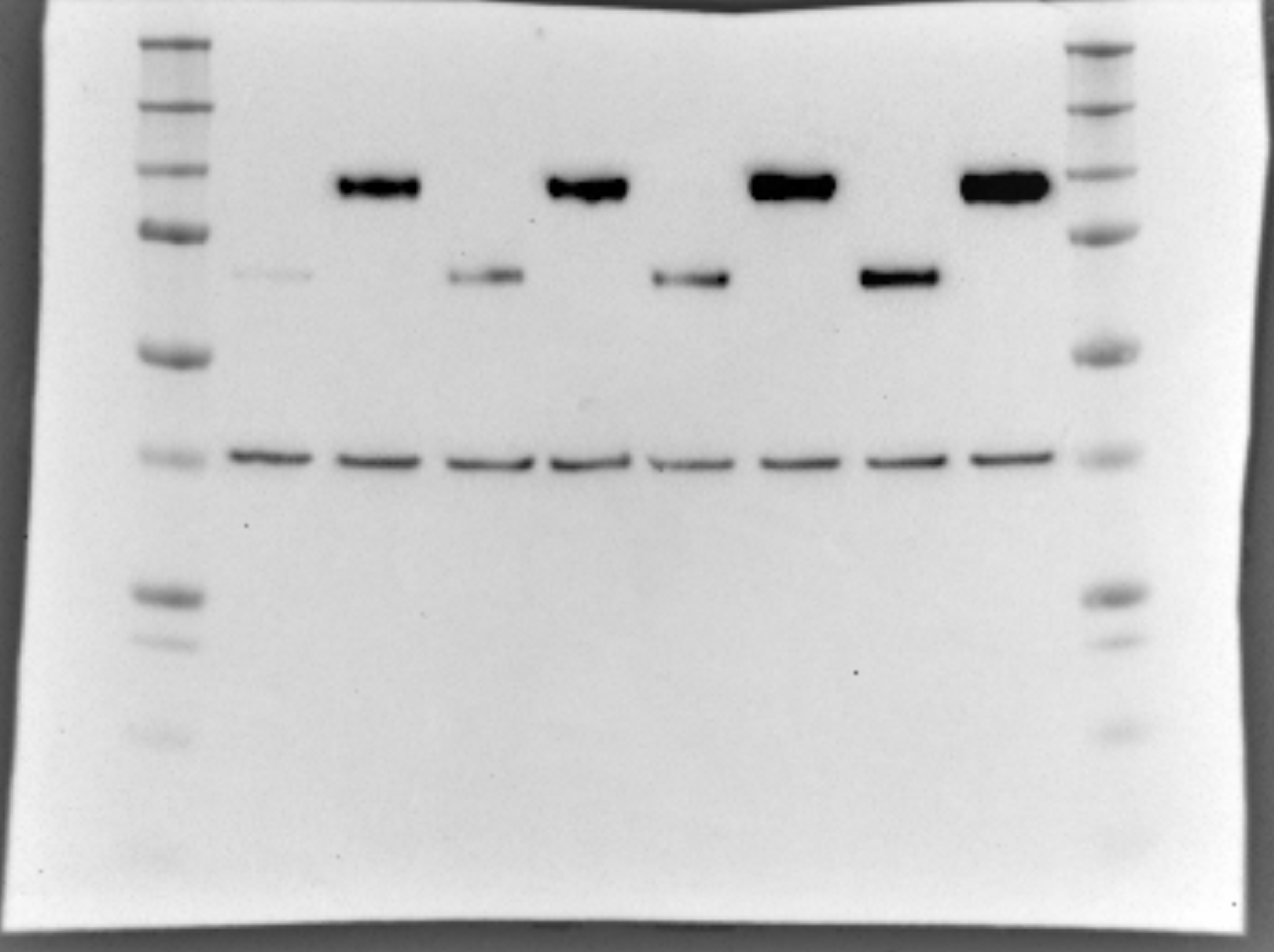


Figure 7C


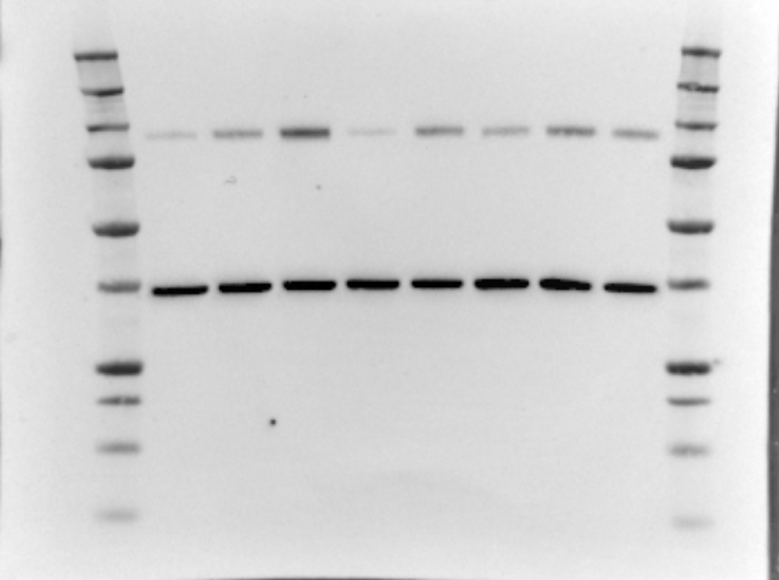


Figure 7D upper panel

***
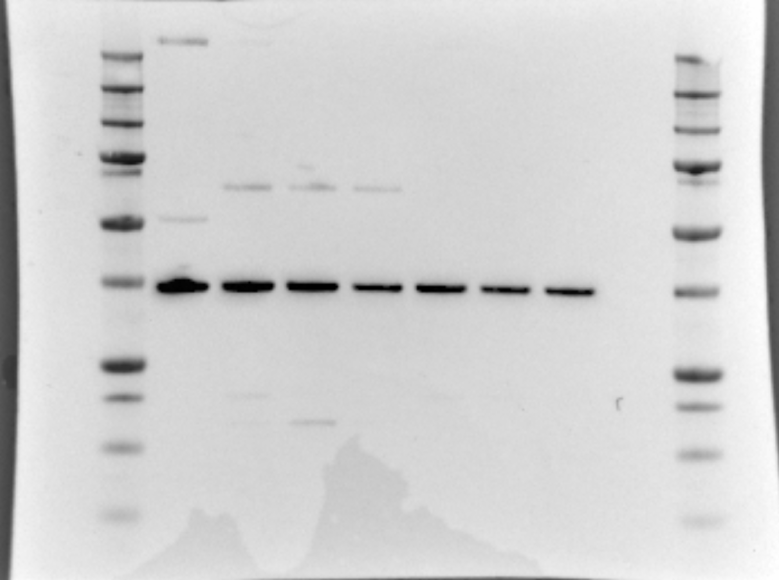
***

Figure 7D lower panel


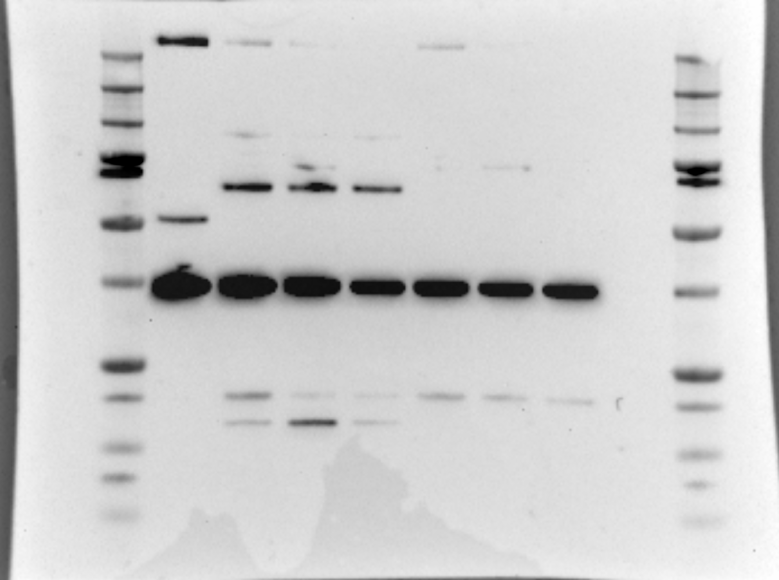

Supplement: Supplementary file 7 — Original blots [file 41419_2026_8926_MOESM7_ESM.docx]
