# Supplementary material for: Selective vulnerability of dopaminergic neurons in Parkinson’s disease connects PRKN and differential expression of CHCHD2 and GPNMB
Source: Cell Death Dis. 2026 Jun 5;17(1):544. doi: 10.1038/s41419-026-08926-4 (PMC13241505; doi:10.1038/s41419-026-08926-4)
Supplement: Supplementary file 8 — Supplemental information [file 41419_2026_8926_MOESM8_ESM.pdf]

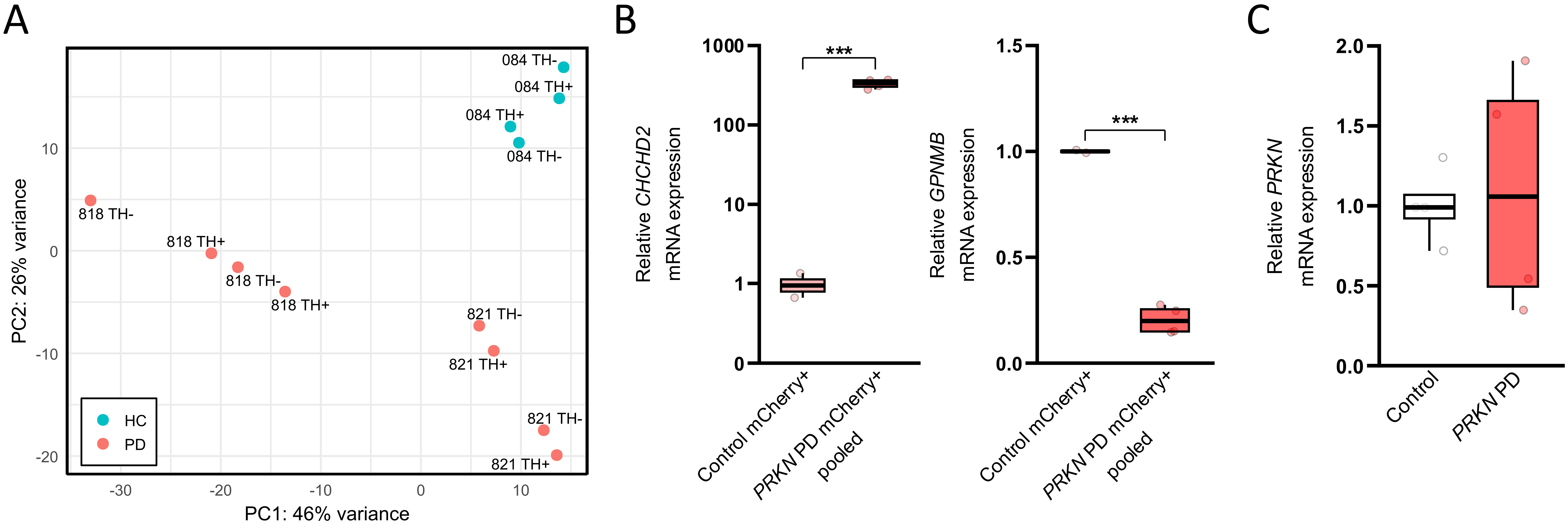

Supplementary Figure 1: RNA sequencing principal component analysis and qRT-PCR validation. (A) Principal component analysis (PCA) scatterplot showing the first and second principal components of RNA-seq samples, demonstrating sample clustering and variability. **(B)** qRT-PCR quantification of *CHCHD2* and *GPNMB* in the sequenced neurons validates the RNA sequencing results shown in Figure 6 (n= 2-4 per group). **(C)** qRT-PCR quantification of *PRKN* mRNA expression in fibroblasts from healthy controls and biallelic *PRKN* mutation carriers. Similar levels indicate a lack of nonsense mediated decay of mutated *PRKN* transcripts in certain carriers, likely explaining why *PRKN* is not differentially expressed on a transcriptional level in *PRKN* mutation carriers compared to controls as shown in Figure 6 (n = 4 per group). p-values <0.05 were considered significant.
